# Acute radiation exposure in mice reveals traces of oxidation-mediated modifications in serum albumin

**DOI:** 10.1371/journal.pone.0330925

**Published:** 2025-09-19

**Authors:** Masaru Yamaguchi, Yota Tatara, Yoshiaki Sato, Ikuo Kashiwakura

**Affiliations:** 1 Hirosaki University Graduate School of Health Sciences, Hirosaki, Japan; 2 Hirosaki University Graduate School of Medicine, Hirosaki, Japan; 3 University of Southern California Norris Comprehensive Cancer Center, Los Angeles, California, United States of America; USUHS: Uniformed Services University of the Health Sciences, UNITED STATES OF AMERICA

## Abstract

High doses of ionizing radiation induce specific oxidation-mediated modifications on the sequence of amino acid that constitutes serum albumin, but the time-dependent response thereafter is unclear. We, therefore, used liquid chromatography coupled with tandem mass spectrometry to profile oxidation-mediated modifications in serum collected from mice subjected to total body X-ray irradiation (TBI) at 20-day intervals up to day 80. In TBI mice, albumin structural regions were generally glycated and oxidatively modified, and in particular, oxidative modifications of serum albumin (OMSA) were observed on either analysis day in eight sequences containing the following amino acid residues: OMSA9 (oxidation of lysine residue (Lys)-97), OMSA12 (oxidation of methionine residue (Met)-147), OMSA21 (oxidation of tyrosine residue (Tyr)-287), OMSA22 (oxidation of proline residue (Pro)-306), OMSA25 (oxidation of Lys-310), OMSA29 (oxidation of Pro-390), OMSA40 (oxidation of Tyr-476), and OMSA39 (nitration of Tyr-476). Interestingly, some significant OMSA sequences induced by TBI were maintained even after 41 times the half-life of albumin. These results suggest that exposure to ionizing radiation induces specific oxidative modifications in serum albumin, and there are specific amino acid residues that are sensitive to these modifications. Furthermore, these could be applied as novel biomarkers to estimate the presence or history of radiation exposure.

## Introduction

Exposure to ionizing radiation can cause direct changes that disrupt atomic structure and indirect changes that damage nucleic acids, lipids, or proteins through the radiolysis of water, ultimately leading to irreversible changes and cell death in healthy cells and tissues [1,2]. Acute radiation syndrome (ARS) is a series of disorders that occur in the acute phase (several days to months) due to whole-body radiation exposure of approximately 1 Gy or more, which may include bone marrow damage, gastrointestinal disorders, skin damage, bleeding, and even death in the worst cases. Clinical symptoms such as vomiting or diarrhea after radiation exposure serve as approximate diagnostic indicators of when and how much radiation was received [3]. However, in reality, the likelihood of an individual being exposed to high or lethal doses of radiation is extremely low, and most radiation exposure is thought to be low or non-lethal levels. The damage caused by radiation exposure progresses gradually and manifests as an increased risk of cancer. The increasing risk of radiation/nuclear exposure highlights the need for functional radiation biomarkers for disaster victims. Radiation biomarkers can be useful in estimating and detecting the extent of radiation exposure, and can also be utilized as triage tools to separate healthy from at-risk populations in mass casualty situations [4–6]. Current practice for estimating radiation exposure using biological samples involves the monitoring of physical symptoms such as nausea/vomiting and peripheral lymphocyte kinetics, or biodosimetry to quantify chromosomal abnormalities in peripheral blood lymphocytes. In particular, chromosomal abnormality analysis is highly specific and sensitive to radiation, but it is not convenient and rapid because it requires a great deal of time and effort to complete the analysis, and is therefore not suitable for formulating treatment plans, predicting prognosis, or allocating emergency medical treatment to victims [7,8].

In light of these limitations, there has been an active search for molecular biomarkers that represent radiation-induced injury in individuals to provide early and rapid diagnosis of victims and to develop methods to complement existing techniques of biological dosimetry [9–11]. In particular, quantitative proteomics is a high-throughput method that can grasp the overall proteome dynamics in cells/tissues/organisms under specific conditions, such as individual disease states or responses to oxidative stress due to radiation exposure. Significant advances in mass spectrometric technology over the last few decades have dramatically improved the ability to accurately identify and quantify proteins, often leading to the discovery of new biomarkers and potential therapeutic targets. We have also used liquid chromatography-tandem mass spectrometry (LC-MS/MS) to search for serum proteins whose expression levels change following radiation exposure [12–14]. In particular, in an analysis of residents living in the Mamuju, Sulawesi, Indonesia, an area with extremely high natural radiation levels (69.6 mSv per year) compared to other parts of the world, we developed a high-resolution multiple reaction monitoring (MRM) method targeting specific oxidative modification of amino acid residues in the constituent sequences of serum albumin, and reported that several amino acid residues are modified via oxidation in a radiation dose-dependent manner, which we call OMSA (oxidative modification of serum albumin) [12,13]. This finding indicated that traces of persistent radiation exposure may be recorded in human serum albumin, and since this is the first analytical method in the world, it provides a unique opportunity to search for novel radiation-induced biomarkers. Furthermore, specific oxidative modifications were observed not only in humans exposed to chronic low-dose radiation but also in the oxidative modification profiling analysis of mouse serum albumin one day after acute high-dose radiation exposure [14]. Reactive oxygen species (ROS) generated in the body cause oxidative modifications of DNA and proteins, and their excessive production and accumulation in various pathological conditions ultimately lead to the breakdown of cellular functions [15]. Albumin is a protein produced mainly in the liver, and accounts for about 60% of the more than 100 types of proteins present in the blood. The half-lives of albumin in the blood are approximately 3 weeks in humans and 35 hours in mice, respectively [16], but it is known that albumin is exposed to various radicals and undergoes post-translational modifications, such as glycation, carbamylation, dimerization, and oxidation. A comprehensive analysis of these modifications of serum albumin circulating in the blood can provide information on the overall oxidative stress experienced by an individual [17]. However, the subsequent dynamics of the oxidative modifications once generated by radiation exposure *in vivo* are unknown.

In this study, serum samples were collected over time from ARS model mice exposed to whole-body radiation, and the time-dependent response of OMSA after radiation exposure and its potential application as a radiation-induced biomarker were examined.

## Materials and methods

### Ethical statement

All procedures related to animal studies were reviewed and approved by the Animal Research Committee of Hirosaki University (approval number: G17001) and were carried out under the Rules for Animal Experimentation of Hirosaki University and the ARRIVE guidelines. In this study, every effort was made to minimize the number and the suffering of animals used in the study and to comply with current animal welfare regulations.

### Experimental design

Forty inbred C57BL/6JJcl female mice, aged 7 weeks, were obtained from CLEA Japan, Inc. (Sendai Branch). Individual identification was controlled by a tattoo drawn on the tail. The mice were housed in sterile polycarbonate cages with stainless steel wire mesh and paper bedding, up to four mice per cage, and were allowed to acclimate to the environment for one week before X-irradiation in a clean vivarium in our facility. The vivarium was set at 23°C in temperature, 50% in humidity, and a daylight-night rotation of 12 hours. Immediately after delivery, the mice were given free access to CLEA Rodent Diet CE-2 and tap water in Hirosaki City as drinking water. The housing environment for the mice was replaced with a new one once a week to keep it clean. Acclimatized mice were assigned to either X-irradiated or non-irradiated groups (20 mice per group). The X-irradiated group was further divided into 5 groups (4 mice per group): D1, analyzed 24 hours; D20, analyzed on the 20^th^ day; D40, analyzed on the 40^th^ day; D60, analyzed on the 60^th^ day; and D80, analyzed on the 80^th^ day after total body irradiation (TBI), as well as 5 corresponding control groups (4 mice per group). This sample size was determined taking into account the limitations of the equipment available at the authors’ institution and the number of analyses that can be performed per run. Mice were monitored once a day at set times to assess their general health, including body weight fluctuations, food intake, activity levels, and hair condition. In particular, X-irradiated mice were classified into the highest distress category, which is likely to involve unavoidable severe stress or prolonged pain, so human endoponts were applied when mice showed symptoms such as distress, abnormal appearance, or a sudden body weight loss of 20% or more. However, no mice were excluded due to human endpoints in this study. After each blood draw (maximum 80 days after radiation exposure), mice were thoroughly anesthetized using isoflurane (Powerful Isoful; Zoetis, London, UK) and then euthanized by cervical dislocation. Different researchers were responsible for mouse housing, X-ray irradiation, sample collection, and analysis.

### X-irradiation experiment

Mice that had been acclimatized for one week after delivery were subjected to a TBI dose of 3.0 Gy of X-rays using an MX-160Labo (MediXtec, Chiba, Japan) set under the following conditions: tube voltage, 160 kV; tube current, 3 mA; dose rate, 0.622 Gy per minute; transparent window filter, 1.0 mm aluminum; focus-to-subject distance, 300 mm. On the other hand, mice in the 5 non-TBI control groups were simply housed in the irradiation apparatus for the same amount of time.

### Evaluation of body hair graying

The degree of graying of mouse body hair due to radiation exposure was analyzed using Image J (https://imagej.net/ij/, last accessed October 1, 2024), an open-source and public-domain image processing software. Five 10 mm × 10 mm regions of interest were randomly set in a straight line along the spine for each individual, and the mean gray value was measured. The average brightness value within the selected region is represented by the mean gray value that ranges from 0 (black) to 255 (white) and is calculated by dividing the sum of gray values by the number of pixels in the region of interest.

### Serum collection

After anesthesia using isoflurane, peripheral blood was collected from the orbital venous plexus of each mouse using a capillary tube on D1, D20, D40, D60, and D80, and left for 1 hour to allow for clotting at room temperature. Capillary tubes were then centrifuged at 1200 × g for 10 min, and the hematocrit values were measured using a hematocrit gauge. The supernatant was collected as a serum component for the following analysis.

### Quantitative measurement of mouse serum albumin

The albumin concentration in serum was analyzed according to the manufacturer’s protocols using LBIS Mouse Albumin enzyme-linked immunosorbent assay (ELISA) kits (FUJIFILM Wako Pure Chemical, Osaka, Japan).

### Serum proteome analysis

Based on the methods of our previous reports [12–14], two microliters of serum were diluted with 198 μl of 50 mM ammonium bicarbonate and precipitated with acetone. The precipitate was resuspended in 20 μl of 50% trifluoroethanol containing 250 mM ammonium bicarbonate and reduced with 40 mM dithiothreitol at 90°C for 30 min. Free cysteine residues were alkylated with 80 mM iodoacetamide for 60 min at room temperature in the dark, and the remaining iodoacetamide was quenched with dithiothreitol. The samples were then diluted with 100 mM ammonium bicarbonate. The samples were incubated with 5 μg trypsin (TPCK-treated, AB Sciex, Framingham, MA, USA) at 37°C for 18 h. The samples were desalted with MonoSpin C18 (GL Sciences). The eluates were dried in a vacuum centrifuge. Desalted samples were rehydrated in 0.1% formic acid and analysed by LC-MS/MS using a nanoLC Eksigent 400 system coupled online to a TripleTOF6600 mass spectrometer (AB Sciex). Peptide separation was performed using a nano C18 reverse-phase capillary tip column (75 μm × 125 mm, 3 μm, Nikkyo Technos Co.) at 300 nl/min with a 90 min linear gradient of acetonitrile in 0.1% formic acid. For protein quantification, data-independent acquisition mode (sequential window acquisition of all theoretical fragment ion spectra mass spectrometry (SWATH) acquisition) was performed. The acquired spectra were processed using DIA-NN software in a label-free and library-free workflow, and semi-quantitative normalized values were obtained.

### Oxidative modification profiling

The same samples prepared for the proteomic analysis were used for OMSA measurements. The LC-MS system was comprised of a high-performance liquid chromatography coupled to a QTRAP6500 + MS (AB Sciex) in electrospray ionization mode. The peptide samples were injected onto a high-performance liquid chromatography C18 column (Aeris PEPTIDE, 2.1 × 100 mm, 2.6 µm, Phenomenex) with a guard column (Aeris PEPTIDE guard cartridge, 2.1 mm, 2.6 µm, Phenomenex) at 40°C using a 10 min solvent gradient employing 0.1% formic acid in water (solvent A) and 0.1% formic acid in acetonitrile (solvent B). Additional LC settings for LC-MS/MS are as follows: 50 to 100% B in 5 min; 100% B in 0.5 min; 100 to 50% B in 0.5 min; 50% B in 1 min at a flow rate of 0.25 ml/min. MS settings for LC-MS/MS mode are as follows: curtain gas, 30; ion spray voltage, 4500 V; temperature, 400°C; ion source gas 1, 50 psi; ion source gas 2, 70 psi; collision gas, 9 psi. OMSAs were identified and quantified using MRM. The values for Q1, Q3, declustering potential, entrance potential, collision energy, and collision cell exit potential for each OMSA in MRM measurements are listed in Supplemental S1 Table. For MRM data analysis, MultiQuant 3.0 Software (AB Sciex) was used to calculate the integrated area of each peptide. All peak selections were checked manually after the automated matches. The peak areas of peptides with oxidative modification were normalized to the unmodified peptides.

### Enrichment analysis using a bioinformatics tool

An orthogonal partial least squares discriminant analysis (OPLS-DA) was performed as a multivariate analysis using the Simca software program (Infocom Co., Tokyo, Japan). OPLS-DA relies on a projection of the data onto the X axis, but it is a supervised modeling approach guiding a maximum separation model. Accordingly, OPLS-DA is suitable for diagnosing differences between two groups or systems to visualize the reliable marker candidates. Further visualization was performed with the help of volcano plots. SRplot (https://www.bioinformatics.com.cn/srplot, last accessed October 1, 2024), a web server that is free and easy to use, was our choice for visualization of the proteomic data. Volcano plots allow visualization of the relationship between fold change (FC) on the horizontal axis and statistical significance on the vertical axis, showing that the expression levels of proteins change significantly as each plot moves away from the graph center. The statistical significance threshold was set at FC > 1.5 or FC < 0.67 with Benjamini-Hochberg’s adjusted P-value (P-value) < 0.05. Red or blue circles mean statistically significant up- or down-regulation, respectively, while open circles indicate no significant change. Gene Ontology (GO) enrichment analysis, focusing on proteins that showed statistically significant expression changes, was performed with the WEB-based GEne SeT AnaLysis Toolkit (WebGestalt, https://www.webgestalt.org/, last accessed October 1, 2024). Example molecular structures of oxidatively modified amino acids were created using the free chemical drawing program BKChem (https://bkchem.zirael.org/, last accessed October 30, 2024).

### Statistical analysis

Significance levels were calculated using the Excel 2019 software program (Microsoft, Redmond, WA, USA) with the Statcel-the Useful Addin Forms on Excel-4th edition (OMS, Tokyo). Comparisons between two groups were performed using the Student’s *t* test for normal distribution and equal variances, the Welch’s *t* test for normal distribution and unequal variances, and the Mann–Whitney *U* test for non-normal distributions, with a *P* value of <0.05 considered to indicate statistical significance.

## Results and discussion

### Basic biological responses after radiation exposure

**Fig 1:**
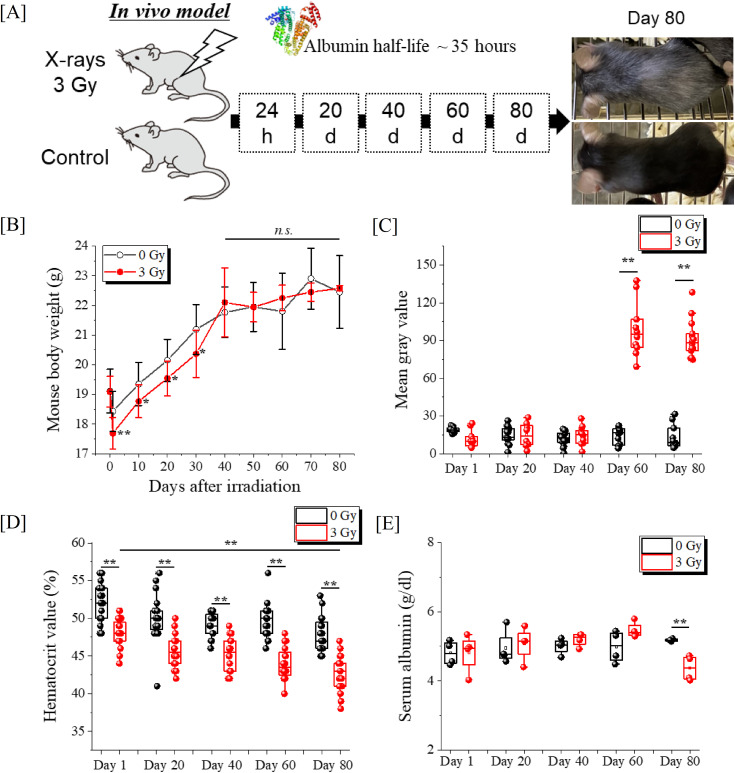
Time course responses in radiation-exposed mice. (A) The serum collection schedule for C57Bl/6JJcl female mice that received 3 Gy TBI. Control (n = 20) and X-irradiated (n = 20) groups were prepared, and four mice from each group were sampled on each collection day. (B) Mouse weight change (g), (C) degree of graying of mouse hair, (D) hematocrit value (%), and (E) serum albumin content were shown. Each plot and vertical bar of the line graph represents the mean ± standard deviation, and box plots show the minimum, first quartile, second quartile, third quartile, and maximum values, with raw data plotted together. Comparisons between two groups were performed using the Student’s *t* test for normal distribution and equal variances, the Welch’s *t* test for normal distribution and unequal variances, and the Mann–Whitney *U* test for non-normal distributions, with a *P* value of <0.05 (*) and <0.01 (**) considered to indicate statistical significance, or not significant (ns).

In our previous report, mice were irradiated with X-rays ranging from 0 Gy to a maximum of 3.0 Gy, and serum collected 24 hours later was analyzed by LC-MS/MS, revealing ionizing radiation dose-dependent reactions of serum proteins, particularly oxidation-mediated modifications on the sequence of albumin amino acid residues. Oxidative modification profiling found significant correlations between the radiation exposure dose and the formation of 11 types of OMSA, including oxidation of the Lys-97 and Tyr-287 [12–14]. In the present study, to investigate how serum proteins and OMSA change over time, serum samples were collected simultaneously from ARS and control mice every 20 days until day 80, which corresponds to 55 times the albumin half-life (Fig 1A). During the first month after TBI, the body weight of the TBI group was significantly lower than that of the non-TBI group, but as the days passed, the body weight increased, and the difference disappeared 40 days after TBI (Fig 1B). In addition, graying of mouse body hair was observed beginning approximately 60 days after TBI, suggesting ectopic differentiation of melanocyte stem cells in their niche located in the bulge region of the hair follicle [18] (Figs 1A and C). Hematocrit values for D1, D20, D40, D60, and D80 were significantly decreased in the TBI group on each blood collection day (Fig 1D). Furthermore, the hematocrit levels of the D80 mice in the TBI group were significantly lower than those at D1. Serum albumin levels were not different between the two groups up to 60 days after TBI, but a significant decrease was observed at D80 in the TBI group (Fig 1E).

### Serum proteome changes due to radiation exposure

**Fig 2:**
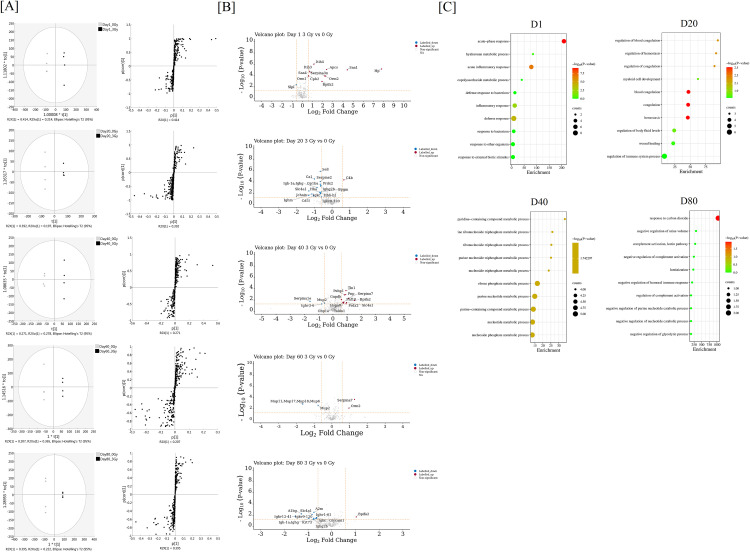
Analysis of serum proteomic change in mice exposed to ionizing radiation. (A) Score scatter plots and S-plots after applying an OPLS-DA to the dataset of each day. Each score scatter plot displays the Hotelling T2 (0.95) range. Black or gray points mean the data of TBI or control mice, respectively. (B) Volcano plots of D1, D20, D40, D60, and D80 showing differentially expressed proteins by radiation. The log_2_(FC) value > 0.585 or log_2_(FC) value <−0.585 with −log_10_(P-value) > 1.3 are the cutoff values for significant up-regulated (red color), non-significant (gray color), and significant down-regulated (blue color) proteins. (C) GO enrichment analysis using variable serum proteins that were significantly differentially expressed by radiation, except D60. Bubble plots of the GO terms in biological processes based on *P*-values are shown. Enrichment is defined as the ratio between the intersection and query size. Counts refer to interaction size, i.e., the number of proteins corresponding to an ontology term.

We examined the impact on the expression of protein and the formation of OMSA in serum collected from ARS and control mice every 20 days until day 80. Instantly, we visualized the reliable marker candidates using a score scatter plot and S-plot after applying an OPLS-DA to the dataset. The score scatter plots in Fig 2A demonstrated good separation between TBI and control mice on D0, D20, D40, D60, or D80, respectively. These results suggest that the profiles of protein expression of TBI and control mice are distinct. Changes in the serum proteome were analyzed over time in individuals exposed to a single acute radiation dose. This analysis revealed that the expression of 323 types of serum proteins was altered by radiation exposure on D1, 323 types on D20, 327 types on D40, 325 types on D60, and 325 types on D80, respectively. Among these, the proteins whose expression was significantly changed by radiation exposure compared to the control group (|log_2_FC| > 0.585 and cutoff *P* value of less than 0.05) were 12 types of proteins on D1 (11 up- and 1 down-regulated), 17 types on D20 (1 up- and 16 down-regulated), 15 types on D40 (12 up- and 3 down-regulated), 4 types on D60 (2 up- and 2 down-regulated), and 12 types on D80 (1 up- and 11 down-regulated), as shown in the volcano plot together with the protein names (Fig 2B, Supplemental S2 Table). The enrichment analysis based on biological process criteria of GO terms was performed on the analysis days other than D60, where there were fewer significantly differentially expressed proteins. These proteins mainly regulate various physiological processes occurring in the body through “regulation of immune response and inflammation,” “protection and repair of cells against inflammation and oxidative stress,” “maintenance of blood and circulatory system function,” and “regulation of cellular functions through glycolysis, cell-cell adhesion, and energy production” (Fig 2C).

### Time-dependent changes of OMSA

**Fig 3:**
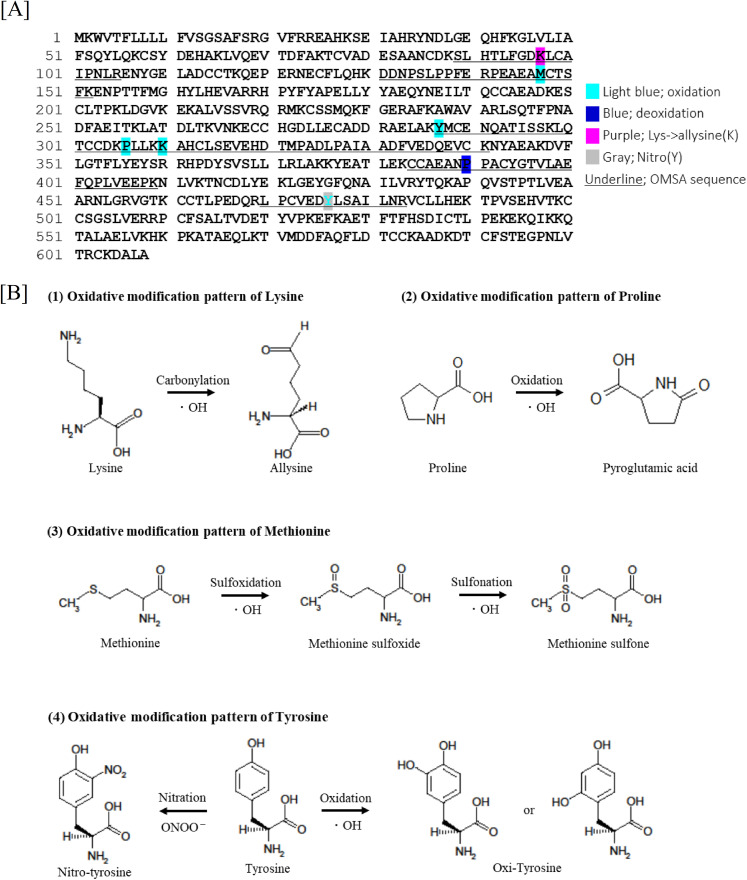
Patterns and sites of oxidation-mediated modification of serum albumin. (A) The patterns and sites of OMSA are highlighted as follows: gray (nitration), purple (carbonylation), blue (di-oxidation), and light blue (oxidation), respectively. The OMSA sequences are underlined. Tyrosine residues can be nitrated by peroxynitrite or oxidized by hydroxyl radicals to give nitrotyrosine or oxi-tyrosine, respectively. Methionine residues can be sulfoxidized by hydroxyl radicals to give methionine sulfoxide, which can then be sulfonated to give methionine sulfone. Lysine or proline residues can be carbonylated or oxidized by hydroxyl radicals to give allysine or pyroglutamic acid, respectively.

Our analysis showed that acute exposure to single radiation led to oxidative changes in albumin’s chemical and spatial structure. The albumin structural region is globally glycosylated and undergoes various oxidative changes, including oxidation of lysine, methionine, proline, arginine, and tyrosine residues, and nitration of tyrosine residues. For the identification of mouse OMSA, peptides containing amino acid residues modified via oxidation were normalized with the peak area values of the corresponding unchanged peptides, and the sequence and modification information of 48 types of OMSA were listed in a previous report [14]. Among these, the following eight sequences, in particular, were found to have been significantly oxidized in ARS mice compared to control mice on either analysis day: OMSA9 (oxidation of Lys-97), OMSA12 (oxidation of Met-147), OMSA21 (oxidation of Tyr-287), OMSA22 (oxidation of Pro-306), OMSA25 (oxidation of Lys-310), OMSA29 (oxidation of Pro-390), OMSA40 (oxidation of Tyr-476), and OMSA39 (nitration of Tyr-476) (Table 1). The temporal relative changes in each oxidative modification pattern due to radiation exposure were also shown in Table 1, whose values were normalized to those in control mice. OMSA9 showed statistically significant oxidative modification on D1 (1.43 ± 0.07), D20 (1.32 ± 0.14), and D60 (1.37 ± 0.22), OMSA21 on D20 (1.22 ± 0.07) and D40 (1.28 ± 0.21), and OMSA39 on D1 (1.42 ± 0.13), D20 (1.32 ± 0.12), and D40 (1.39 ± 0.19) compared to the control mice across multiple analysis dates. On the other hand, OMSA12 showed significant oxidative modifications on D20 (1.19 ± 0.12), OMSA22 and OMSA25 on D40 (4.37 ± 2.24 and 1.17 ± 0.03, respectively), and OMSA29 and OMSA40 on D1 (1.29 ± 0.09 and 1.66 ± 0.63, respectively) compared to the control mice on a single analysis date. By 60 days after irradiation, all OMSAs except OMSA9 had returned to the same level as the control, and by 80 days after irradiation, OMSA9 was also at the same level as the control. The modification sites identified on the amino acid sequence of mouse albumin are highlighted (Fig 3A). Proteins undergo various chemical changes, mainly through the side chains of their amino acid residues, that react with ROS. ROS generated in the body cause oxidative modifications of DNA and proteins, and their accumulation in various pathological conditions ultimately leads to the breakdown of cellular functions [19]. Proteins contain many amino acid residues that are easily oxidized, and when the concentrations of reactive chemical species such as hydroxyl radicals, peroxynitrite, and nitric oxide increase in the body, they undergo various oxidative modifications [20,21]. Regarding protein oxidation, carbonylation of the amino acid lysine residues and oxidation of methionine residues are well known [22]. In particular, albumin, which is the most abundant component in serum [23], is frequently exposed to a variety of reactive chemicals and may therefore reflect the oxidative stress state of the entire body. In this study, radiation exposure in mice was also confirmed to cause the oxidation of lysine residues to allysine (2-aminoadipic semialdehyde), which is an indicator of oxidative stress and oxidized proteins that increase with age, and the oxidation of the pyrrolidine ring of proline residues to pyroglutamic acid (Fig 3B). When superoxide radicals and nitric oxide coexist in vivo, they react to produce peroxynitrite, which has a stronger oxidizing power and higher cytotoxicity. Peroxynitrite, in particular, reacts with the phenolic ring of tyrosine residues to generate nitrotyrosine, a nitration modification (Fig 3B). Tyrosine nitration is widely used as a nitrative stress marker, and has been reported to be associated with numerous diseases, including neurological disorders such as Alzheimer’s disease, Parkinson’s disease, multiple sclerosis, and stroke, as well as atherosclerosis, myocardial infarction, coronary artery disease, and hypertension. [24]. Furthermore, the methyl thioether group in methionine residues is converted to a sulfoxide structure and finally to a methyl sulfone structure in response to increased intracellular oxidative stress.. Still, the majority of methionine observed in cells is the sulfoxide type (Fig 3B). An increase in methionine sulfoxide has been observed in certain proteins in the tissues of aged animals. Since hair graying due to radiation exposure was observed (Fig 1C), it is highly likely that it is involved in the decline in function associated with aging [25]. Interestingly, although the half-life of mouse albumin *in vivo* is about 35 hours, significant oxidative modifications of specific amino acid sequences generated by radiation exposure were observed even after 41 times the half-life of albumin. Aging-related decline in proteasome activity causes the accumulation and aggregation of abnormal proteins [26], which is believed to be involved in the late onset and progression of neurodegenerative diseases, and it is thought that the process of breaking down and recycling abnormal proteins is suppressed by radiation exposure. In addition, GO analysis using serum proteome data from D20 extracted GO terms such as “regulation of hemostasis” and “myeloid cell development” (Fig 2C), and the proportion of red blood cells in the blood was significantly reduced in the irradiated group (Fig 1D). In response to the decrease in peripheral blood cells after radiation exposure, hematopoietic stem cells induce sustained increased division activity, which promotes aging, and the decrease in antioxidant capacity due to aging leads to the accumulation and delayed production of ROS [27–29], which may contribute to the persistence of OMSA. Some sites capture the effects of radiation exposure even about 2 months after exposure, and serum albumin content did not change with radiation exposure or time (Fig 1E). Therefore, modified proteins may be a novel indicator that reflects related pathologies and abnormal conditions in the body due to oxidative stress. According to this study, TBI causes complex fluctuations in OMSA that do not diminish depending on the half-life of serum albumin. There may be sequences (amino acid residues) that are particularly sensitive to radiation exposure. This may reflect the oxidative stress response and the diverse functions of serum albumin in vivo, which is important when considering the long-term effects of radiation injury. On the other hand, in our previous work on LC-MS/MS analysis of residents in an area with high natural radiation levels in Indonesia, we reported that chronic low-dose radiation-dependent oxidation was induced in specific amino acid residues in the human albumin sequence [12,13]. Oxidative modifications have also been observed in specific amino acid residues in the mouse albumin sequence in a mouse model exposed to acute high-dose radiation [14], but these profiles of OMSA, likely due to different doses, routes, and forms of radiation exposure, differ significantly between mice and humans. However, it is common for radiation exposure to induce specific OMSA formation in both humans and mice, and traces of these modifications may continue to remain even after radiation exposure, so the results obtained in this mouse model may be generalizable to human biology. In conclusion, although the experimental conditions in this study were limited, such as the dose and type of radiation, we showed the possibility that oxidative modification profiling of serum albumin following acute high-dose radiation exposure could be applied as a high-throughput and prospective radiation-induced biomarker. This may be an attractive and novel strategy for rapid triage of people at risk of developing ARS in radiation emergencies, but it remains future challenges to clarify the usefulness of OMSA as a radiation-induced biomarker, including functional changes of serum albumin by oxidation, its effects on the organism itself, and its relationship with ARS.

**Table 1:** Temporal relative change of each OMSA after exposure to total-body irradiation.

OMSA	Amino acid residue	Modification	D1		D20		D40		D60		D80
09	Lys-97	Carbonylation	1.43 ± 0.07	*	1.32 ± 0.14	*	1.09 ± 0.12		1.37 ± 0.22	*	1.01 ± 0.04
12	Met-147	Oxidation	1.03 ± 0.26		1.19 ± 0.12	*	1.25 ± 0.17		1.13 ± 0.26		0.99 ± 0.20
21	Tyr-287	Oxidation	1.30 ± 0.37		1.22 ± 0.07	*	1.28 ± 0.21	*	1.11 ± 0.34		1.12 ± 0.14
22	Pro-306	Oxidation	1.50 ± 0.75		0.97 ± 0.20		4.37 ± 2.24	*	1.63 ± 0.97		0.95 ± 0.39
25	Lys-310	Carbonylation	0.86 ± 0.13		1.14 ± 0.07		1.17 ± 0.03	*	1.21 ± 0.31		0.92 ± 0.19
29	Pro-390	Oxidation	1.29 ± 0.09	*	0.93 ± 0.31		2.35 ± 2.34		2.50 ± 2.52		1.60 ± 1.36
39	Tyr-476	Nitration	1.42 ± 0.13	*	1.32 ± 0.12	*	1.39 ± 0.19	*	1.88 ± 1.20		1.18 ± 0.44
40	Tyr-476	Oxidation	1.66 ± 0.63	*	0.84 ± 0.12		1.89 ± 0.93		1.56 ± 0.63		0.89 ± 0.35

*Note*. Comparison of each OMSA between 0Gy and 3Gy groups on any of the analysis dates were performed using the Student’s *t* test for normal distribution and equal variances, the Welch’s *t* test for normal distribution and unequal variances, and the Mann–Whitney *U* test for non-normal distributions, with a *P* value of <0.05 (*) considered to indicate statistical significance. Temporal changes in each OMSA due to radiation exposure, normalized with non-irradiated controls. The values represent the mean ± standard deviation.

## Supporting information

S1 Table The list of MRM conditions. (XLSX)

S2 Table The output of the analysis underlying the volcano plots. (XLSX)

## Abbreviations:

ARS: Acute radiation syndrome
ELISA: Enzyme-linked immunosorbent assay
GO: Gene ontology
LC-MS/MS: Liquid chromatography-tandem mass spectrometry
MRM: multiple reaction monitoring
OMSA: Oxidative modification of serum albumin
OPLS-DA: Orthogonal partial least square-discriminant analysis
ROS: Reactive oxygen species
TBI: Total body irradiation
